# Glycan-Modified Apoptotic Melanoma-Derived Extracellular Vesicles as Antigen Source for Anti-Tumor Vaccination

**DOI:** 10.3390/cancers11091266

**Published:** 2019-08-28

**Authors:** Sophie K. Horrevorts, Dorian A. Stolk, Rieneke van de Ven, Myrthe Hulst, Bert van Het Hof, Sanne Duinkerken, Marieke H. Heineke, Wenbin Ma, Sophie A. Dusoswa, Rienk Nieuwland, Juan J. Garcia-Vallejo, Arjan A. van de Loosdrecht, Tanja D. de Gruijl, Sandra J. van Vliet, Yvette van Kooyk

**Affiliations:** 1 Amsterdam UMC, Department of Molecular Cell Biology and Immunology, Amsterdam Infection and Immunity Institute, Cancer Center Amsterdam, Vrije Universiteit Amsterdam, 1081 HV Amsterdam, The Netherlands; 2 Amsterdam UMC, Department of Medical Oncology, Cancer Center Amsterdam, Vrije Universiteit Amsterdam, 1081 HV Amsterdam, The Netherlands; 3 Amsterdam UMC, Department of Otolaryngology/Head and Neck Surgery, Cancer Center Amsterdam, Vrije Universiteit Amsterdam, 1081 HV Amsterdam, The Netherlands; 4 Ludwig Institute for Cancer Research, B-1200 Brussels, Belgium; 5 Walloon Excellence in Life Sciences and Biotechnology, B-1200 Brussels, Belgium; 6 De Duve Institute, Université Catholique de Louvain, B-1200 Brussels, Belgium; 7 Amsterdam UMC, Laboratory of Experimental Clinical Chemistry, and Vesicle Observation Centre, Academic Medical Centre, 1081 HV Amsterdam, The Netherlands; 8 Amsterdam UMC, Department of Hematology, Cancer Center Amsterdam, Vrije Universiteit Amsterdam, 1081 HV Amsterdam, The Netherlands

**Keywords:** dendritic cell, apoptotic extracellular vesicles, T cell priming, cancer vaccine, glycan modification, C-type lectin

## Abstract

Tumors that lack T cell infiltration are less likely to respond to immune checkpoint inhibition and could benefit from cancer vaccination for the initiation of anti-tumor T cell responses. An attractive vaccine strategy is in vivo targeting of dendritic cells (DCs), key initiators of antigen-specific T cell responses. In this study we generated apoptotic tumor cell-derived extracellular vesicles (ApoEVs), which are potentially an abundant source of tumor-specific neo-antigens and other tumor-associated antigens (TAAs), and which can be manipulated to express DC-targeting ligands for efficient antigen delivery. Our data demonstrates that by specifically modifying the glycocalyx of tumor cells, high-mannose glycans can be expressed on their cell surface and on extracellular vesicles derived after the induction of apoptosis. High-mannose glycans are the natural ligands of dendritic cell-specific intercellular adhesion molecule-3-grabbing non-integrin (DC-SIGN), a dendritic cell associated C-type lectin receptor (CLR), which has the ability to efficiently internalize its cargo and direct it to both major histocompatibility complex (MHC)-I and MHC-II pathways for the induction of CD8^+^ and CD4^+^ T cell responses, respectively. Compared to unmodified ApoEVs, ApoEVs carrying DC-SIGN ligands are internalized to a higher extent, resulting in enhanced priming of tumor-specific CD8^+^ T cells. This approach thus presents a promising vaccination strategy in support of T cell-based immunotherapy of cancer.

## 1. Introduction

Immune checkpoint inhibitors, like anti-PD1 and anti-CTLA-4 blocking antibodies, have improved the long-term survival of patients with metastatic melanoma [1,2]. However, not all patients experience clinical benefit from checkpoint inhibitor therapy [1,2,3,4]. Patients who do not have T cell infiltration in the tumor microenvironment, those who carry so–called “cold tumors”, are less likely to respond and could benefit from tumor vaccination to initiate anti-cancer immunity [5]. The induction of anti-tumor T cell responses relies on the presentation of antigens by dendritic cells (DCs). After antigen capture in the periphery, DCs mature and migrate via the lymphatic system to the lymph nodes. There, DCs present captured antigens via the conventional antigen presentation route on major histocompatibility complex (MHC) class II and via cross-presentation on MHC-I molecules, and combined with appropriate co-stimulation, subsequently prime and activate CD4^+^ and cytotoxic CD8^+^ T cells (CTLs), respectively [6,7]. Efficient delivery of antigens to DCs is key for the induction of tumor-specific T cell responses, and DC-specific C-type-lectin receptors (CLRs) are widely studied as targets for specific antigen delivery [8,9,10]. CLRs endocytose glycosylated pathogens via their carbohydrate recognition domain, allowing for efficient processing of pathogens and antigen loading onto MHC-I and MHC-II molecules [11]. DEC205 was one of the first CLRs used for DC targeting by conjugating antigens to a DEC205-specific antibody (Ab) and, combined with the administration of anti-CD40 for DC activation, resulted in increased CD8^+^ T cell activation and growth inhibition of established B16-OVA tumors in vivo [12,13]. Since CLRs are abundantly expressed on multiple DCs subsets and glycans are their natural ligands, the use of glycan motifs for the targeting of vaccines to DCs is an attractive immunization strategy [10,14]. Dendritic cell-specific intercellular adhesion molecule-3-grabbing non-integrin (DC-SIGN), which is expressed on monocyte-derived DCs (moDCs), and on skin and lymph node resident antigen presenting cell (APCs) subsets [10,15,16,17], is a CLR specific for Lewis-type and high-mannose glycans [18]. After internalization, it efficiently directs its cargo to MHC-I and MHC-II, resulting in enhanced CD8^+^ and CD4^+^ T cell responses when targeted with Lewis^Y^ or high-mannose containing liposomes or proteins [10,19].

Besides efficient DC loading, the choice of antigen is pivotal in anti-cancer vaccine development. There is compelling evidence that the presence of neo-antigens is important in the initiation of tumor-specific T cell responses. Tumors with a high mutational load are associated with increased T cell responses and improved clinical outcome [20,21]. Moreover, vaccination with neo-antigens derived from murine melanoma and colon carcinoma models resulted in tumor control in mice [22,23]. The first neo-antigen-based vaccines clinically tested in melanoma patients also showed promising results with relatively high response rates [24,25]. Neo-antigens are therefore regarded as an attractive vaccine target for the induction of superior anti-tumor T cell responses, but require laborious and costly production pipelines involving genome-wide sequencing, non-synonymous mutation identification, epitope prediction, and RNA or peptide production [24,25]. An abundant source of both patient-specific neo-antigens and shared tumor-associated antigens (TAAs) for vaccination purposes, without the need for prior knowledge of the personalized epitope repertoire, is the autologous tumor itself. We previously showed that apoptotic extracellular vesicles (ApoEVs) derived from tumor cells can induce antigen-specific T cell responses both in vitro and ex vivo [26,27]. ApoEVs are produced when cells undergo apoptosis, resulting in membrane blebbing, membrane protrusion, and apoptotic body (ApoBD) formation [28]. ApoBDs have a diameter of 1–5 μM, express phosphatidylserine (PtdSer) on their membrane surface, and contain, among others, proteins and nuclear fragments [28,29,30,31,32,33]. Besides ApoBDs, apoptotic cells induce formation of smaller vesicles (<1 μM), called apoptotic microvesicles [28,30,31]. In the literature, no consensus is reached regarding the nomenclature of the smaller apoptotic vesicles; therefore, we decided to use the general term ApoEVs to describe all sizes of extracellular vesicles derived from apoptotic cells.

In this study, we designed a DC-targeted tumor vaccine through glycan modification of melanoma-derived ApoEVs. The induction of DC-SIGN-binding high-mannose glycans on tumor cells prior to the induction of apoptosis with bortezomib resulted in high-mannose expressing ApoEVs, which facilitate enhanced uptake by moDCs via DC-SIGN and enabled antigen-specific CD8^+^ T cell priming. These data support the use of glycan-modified ApoEVs as vehicles for the selective in vivo delivery of tumor-derived (neo-)antigens to DCs in order to kick-start tumor-specific CD8^+^ T cell responses.

## 2. Results

### 2.1. Kifunensine-Treated Melanoma Cells Express High-Mannose Carbohydrate Structures on Their Cell Surface

**Figure 1:**
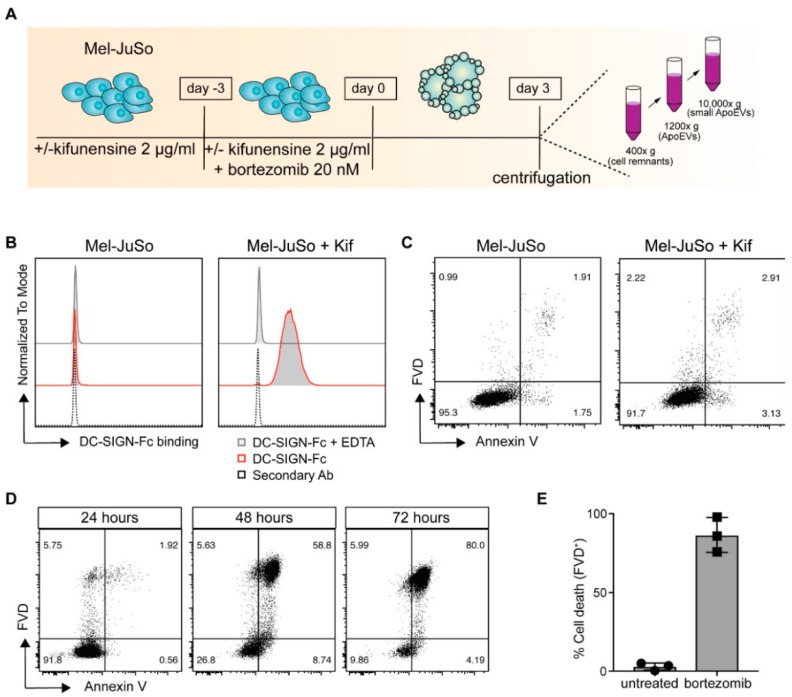
Characterization of kifunensine-treated Mel-JuSo cells. (**A**) Protocol of ApoEV and ApoEV-HM production and isolation procedure. (**B**) Mel-JuSo cells were cultured with kifunensine for 72 h. Dendritic cell-specific intercellular adhesion molecule-3-grabbing non-integrin (DC-SIGN) binding was assessed using DC-SIGN-Fc and measured by flow cytometry. (**C**) Cell viability was measured by flow cytometry after staining with Annexin V and fixable viability dye (FVD). Representative of *n* = 3. (**D**) Mel-JuSo cells were treated with bortezomib to induce apoptosis. After 24, 48, and 72 h Annexin V (as a measure of apoptosis) and the cell viability (FVD) was measured by flow cytometry. Representative plots of *n* = 3. (**E**) After 72 h of apoptosis induction, cell viability ranged between approximately 5–25%. Data shown as mean ± SD of three individual experiments.

To target tumor-specific antigens to moDCs for the induction of superior CTL-responses, we aimed to modify ApoEVs with DC-SIGN binding ligands, such as high-mannose glycan structures (Figure 1A). For the generation of high-mannose expressing ApoEVs (ApoEVs-HM), the melanoma cell line Mel-JuSo was cultured 72 h prior to and during the induction of apoptosis, in the presence of the mannosidase I inhibitor kifunensine, an alkaloid and potent inhibitor of the enzyme mannosidase I [34]. By blocking the trimming of mannose residues on precursor glycoproteins, tumor cells express high-mannose containing glycoproteins [19].

Kifunensine treatment induced expression of DC-SIGN binding ligands, as shown by an increased DC-SIGN-Fc binding to Mel-JuSo cells. This is in concordance with previous work where we showed the expression of DC-SIGN binding ligands on a variety of melanoma cell lines after kifunensine treatment [19]. The enhanced DC-SIGN binding was completely abrogated in the presence of EDTA, thereby confirming the specific binding of the DC-SIGN-Fc molecules, as DC-SIGN binding is Ca^2+^ dependent [35] (Figure 1B). The kifunensine treatment did not affect the viability of the cells (Figure 1C). Mel-JuSo cells were treated 72 h with 20 nM bortezomib to induce the formation of early and late ApoEVs. We selected bortezomib for the generation of ApoEVs, as this compound is already used in the clinic for the treatment of multiple myeloma and B cell lymphoma, and potently induces immunogenic cell death [36,37]. Apoptosis induction was monitored every 24 h by membrane staining of PtdSer (Annexin V) in combination with a viability dye (Figure 1D). We observed, 48 h after the induction of apoptosis, an increase in Annexin V staining and decrease in cell viability, with a cell viability below 25% after 72 h (Figure 1E). The ApoEVs were finally isolated using differential centrifugation steps (400× *g* and 1200× *g*) and collected after centrifugation at 10,000× *g* [32,33].

### 2.2. Glycan Modification Results in ApoEVs with DC-SIGN Binding Properties

**Figure 2:**
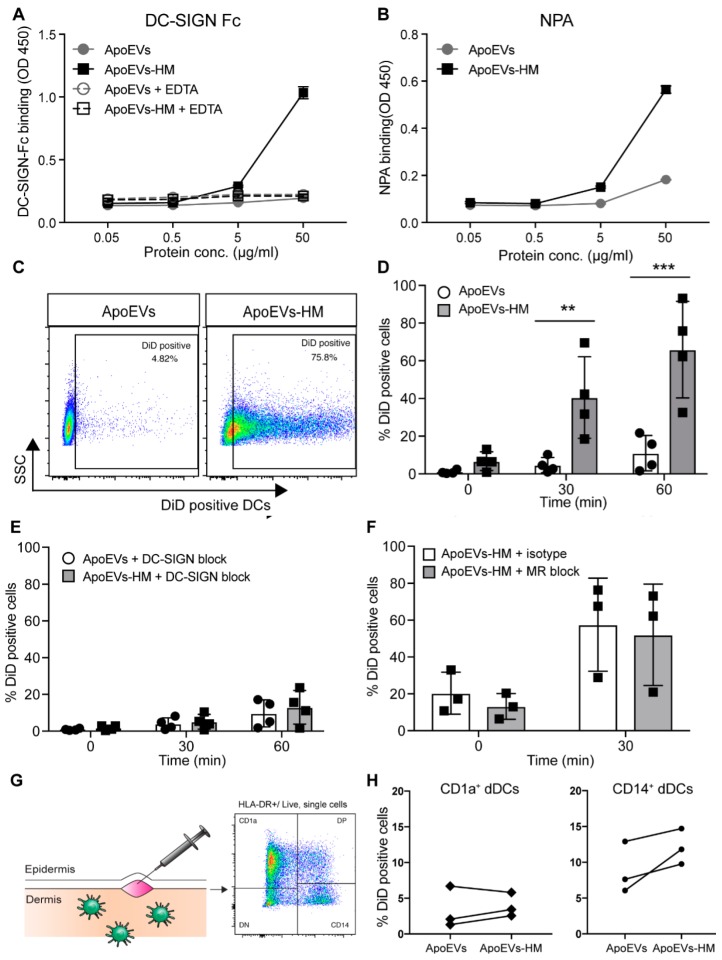
The expression of high-mannose glycans on ApoEVs mediates DC-SIGN binding. (**A**,**B**) Mel-JuSo-derived ApoEVs and ApoEVs-HM were coated, and DC-SIGN binding (**A**) and NPA binding (**B**) were detected by DC-SIGN-Fc or the lectin Narcissus pseudonarcissus agglutinin (NPA), respectively. Data shown as mean ± SD; representative of three individual experiments. (**C**–**F**) MoDCs were pulsed with DiD-labeled ApoEVs and ApoEVs-HM for 45 min at 4 °C, before cells were transferred to 37 °C. Uptake was measured by flow cytometry. (**C**) Representative flow cytometry data at time point 60 min. (**D**) Uptake of ApoEVs and ApoEVs-HM by moDCs after 0, 30, and 60 min. Data shown as mean ± SD of four donors. Statistics performed; two-way repeated measures ANOVA with Sidak post-hoc test. ** *p* < 0.01, *** *p* < 0.001. (**E**) MoDCs were blocked with a DC-SIGN blocking Ab (AZN-D1) or (**F**) MR blocking Ab 30 min prior to the loading with the ApoEVs or ApoEVs-HM. Data shown as mean ± SD of four donors (**E**) or three (**F**) donors. Statistics performed; two-way repeated measures ANOVA with Sidak post-hoc test. (**G**) Gating strategy for the CD1a^+^ and CD14^+^ dermal DCs (dDCs). (**H**) Uptake of DiD-labeled ApoEVs and ApoEVs-HM by migrated dDCs following in situ injection.

We next proceeded to analyze the binding of the different ApoEV and ApoEV-HM batches by DCs. No differences in DC binding could be detected between the unmodified ApoEVs isolated at 1200× *g* or at 10,000× *g*. However, the binding of ApoEVs-HM isolated at 10,000× *g* was significantly increased, compared to the larger vesicles isolated at 1200× *g* (Figure S1). Therefore, we decided to further investigate the immune stimulatory properties of the ApoEVs and ApoEVs-HM isolated at 10,000× *g*. The size distribution of these vesicles was heterogeneous, ranging from approximately 200 nm up to >1000 nm (Figure S2). We checked whether the DC-SIGN binding ligands induced by the kifunensine treatment on the Mel-JuSo cells were preserved on the membranes of the ApoEVs. The vesicles were coated on an ELISA plate and stained with DC-SIGN-Fc (Figure 2A). ApoEVs expressing high-mannose (ApoEVs-HM) bound DC-SIGN-Fc in a dose-dependent manner, in contrast to the unmodified ApoEVs that did not possess DC-SIGN binding properties. The enhanced DC-SIGN binding was completely abrogated in the presence of EDTA, confirming the specificity for DC-SIGN-Fc. Binding of the lectin Narcissus pseudonarcissus agglutinin (NPA) indicated the presence of α1–6 polymannose structures on the high-mannose vesicles (Figure 2B). Next, we generated ApoEVs and ApoEVs-HM from the melanoma cell line SK-Mel28 according to the protocol described in Figure 1A. The SK-Mel28-derived ApoEVs-HM also contained DC-SIGN-binding ligands as detected with DC-SIGN-Fc ELISA (Figure S3), demonstrating that this method can be applied to different tumor cell lines for the generation of DC-SIGN-binding ApoEVs.

### 2.3. High-Mannose Expressing ApoEVs Are Internalized via DC-SIGN by moDCs

To evaluate the DC-SIGN-binding properties of our ApoEVs-HM, we pulsed moDCs with DiD-labeled vesicles for 45 min on ice, before transferring them to 37 °C for an additional 30- or 60-min incubation. The percentage of DiD-positive moDCs was determined as a measure of vesicle binding/uptake. After 60 min at 37 °C, up to 93% of the ApoEV-HM pulsed moDCs were DiD-positive compared to approximately 20% of the moDCs pulsed with the control ApoEVs (Figure 2C,D). Pre-treatment with AZN-D1, a DC-SIGN blocking Ab, completely abrogated uptake of ApoEVs-HM (Figure 2E), showing that the enhanced uptake was completely DC-SIGN-dependent. Because the mannose receptor (MR) on moDCs might also bind mannose structures, we tested whether moDCs could bind ApoEV-HM via MR (Figure 2F). The uptake of ApoEVs-HM was not affected by blocking the MR and was comparable to the uptake of ApoEVs-HM by isotype control treated moDCs. To further investigate the DC-SIGN-targeting properties of ApoEVs-HM, we used a human skin explant model [17], where we injected the vesicles to confirm binding of the ApoEVs-HM to human dermal DCs (dDCs) that naturally express DC-SIGN [16,17]. After two days, the migrated dDCs were analyzed by flow cytometry to identify vesicle uptake (DiD-labeled) by CD1a^+^ (HLA-DR^+^/CD1a^+^) and CD14^+^ (HLA-DR^+^/CD14^+^) dDCs and Langerhans cells (HLA-DR^+^/CD1a^high^/EpCAM^+^) [17] (Figure 2G). A trend of increased ApoEV-HM uptake could be observed in the CD14^+^ dDCs population compared to the CD1a^+^ dDCs subset, which is in line with the higher expression of DC-SIGN on the CD14^+^ subset [16] (Figure 2H). The number of migrated Langerhans cells was, unfortunately, too low to be analyzed.

**Figure 3:**
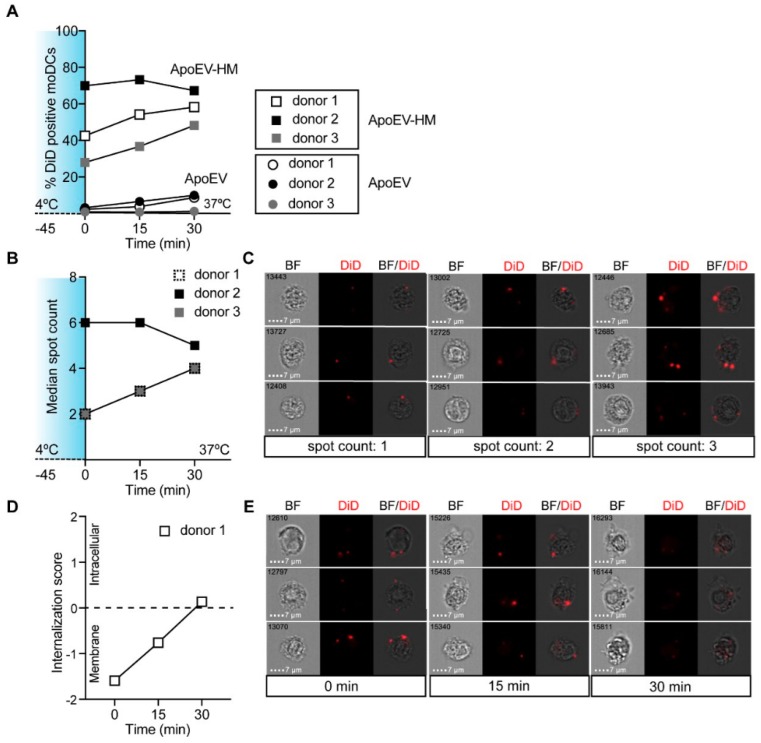
Glycan modified ApoEVs (ApoEVs-HM) are internalized by moDCs. (**A**) MoDCs (white = donor 1, black = donor 2, grey = donor 3) were loaded with DiD-labeled ApoEVs and ApoEVs-HM for 45 min at 4 °C, washed, and incubated at 37 °C for the indicated times. MoDCs were analyzed using imaging flow cytometry. (**B**) Time course of the median spot count in the DiD-positive ApoEV-HM-loaded moDC population using imaging flow cytometry. (**C**) Representative pictures of ApoEV-HM-loaded moDCs at time point 0 min with 1, 2, or 3 positive spots. (**D**) Time course of the internalization score of ApoEVs-HM of donor 1. (**E**) Representative imaging flow cytometry pictures after 0, 15, and 30 min incubation at 37 °C of moDCs (donor 1) that are positive for 2 spots.

Since adjuvants can affect the internalization capacity of moDCs [38], we investigated the DC-SIGN-mediated uptake of ApoEVs-HM in the presence of a TLR4-stimulus (Figure S4). MoDCs that received a TLR-4 stimulus (MPLA) showed similar internalization capacity compared to unstimulated moDCs, demonstrating that DC-SIGN is a suitable receptor for vaccine targeting strategies of both immature and maturing moDCs. To investigate if the ApoEVs were internalized by moDCs, we assessed vesicle uptake by imaging flow cytometry. After 30 min incubation at 37 °C, around 50–65% of the moDCs loaded (at 4 °C) with ApoEVs-HM were positive for DiD (Figure 3A). The amount of ApoEV-positive moDCs that could be used for image analysis was low, therefore we only determined the uptake in ApoEV-HM-loaded moDCs. Using imaging flow cytometry, we could discriminate between membrane-bound and internalized DiD-positive vesicles. We created a mask that we applied to all DiD-positive moDCs, which allowed for counting DiD-positive spots, representing the ApoEVs-HM localized at the membrane surface or intracellular (Figure 3B). After 45 min incubation at 4 °C (time point: 0 min), the DiD-labeled ApoEVs-HM were solely localized at the membrane surface of moDCs (Figure 3C). The internalization score was calculated, as the intensity of the DiD signal of the intracellular space versus the intensity of the entire cell. MoDCs that had internalized ApoEVs-HM typically had positive scores, whereas moDCs displaying membrane-bound ApoEVs-HM had negative scores. (NB: the internalization score was 0 when similar amounts of vesicles were located at the membrane, as in the intracellular space). After 30 and 60 min incubation at 37 °C, the ApoEVs-HM were internalized as indicated by the increase in internalization score (Figure 3D,E representative pictures). Collectively, these data show that DC-SIGN facilitates increased binding and internalization of ApoEVs-HM by moDCs.

### 2.4. Mel-JuSo-Derived ApoEVs Do Not Mature moDCs

**Figure 4:**
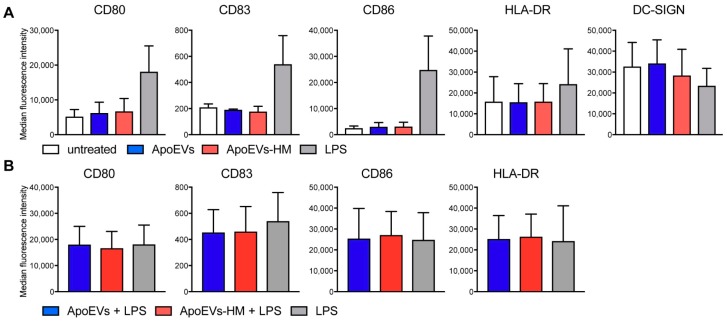
Glycan-modified ApoEVs (ApoEVs-HM) do not mature moDCs. (**A**) MoDCs were loaded with ApoEVs, ApoEVs-HM (100 μg/mL (two donors) or 200 μg/mL (two donors)), or LPS for 3 h, washed, and cultured o/n. The next day, the expression of maturation markers CD80, CD83, CD86, HLA-DR, and DC-SIGN was analyzed by flow cytometry. Graph is combined data of the four donors, showing mean + SD of the median fluorescence intensity. (**B**) MoDCs were loaded with ApoEVs, ApoEVs-HM (100 μg/mL (two donors) or 200 μg/mL (two donors)) in the presence of LPS (10 ng/mL) for 3 h. The next day, the expression of CD80, CD83, CD86, and HLA-DR was analyzed by flow cytometry. Graph is combined data of the four donors, showing mean + SD of the median fluorescence intensity.

Stress-induced apoptosis can result in immunogenic cell death, a form of cell death that facilitates T cell-mediated immune responses and is characterized by the secretion and cell surface expression of damage-associated molecular patterns (DAMPs), like high mobility group protein B1 (HMGB1), heat-shock proteins, and calreticulin [36]. These molecules have the ability to induce DC maturation by binding pattern recognition receptors (PRRs) [37,39]. The presence of DAMPs on the vesicle membrane would allow for self-adjuvating properties of the vesicle vaccine. We investigated the capacity of our isolated vesicles to mature moDCs by quantifying DC maturation markers by flow cytometric analysis. While TLR4 stimulation with lipopolysaccharide (LPS) induced DC maturation as expected, expression levels of CD80, CD86, and HLA-DR did not differ between untreated and vesicle-loaded moDCs (Figure 4A). However, both LPS-stimulated and ApoEV-HM-loaded moDCs appeared to have slightly lower DC-SIGN expression (Figure 4A). These results can be explained by the fact that DC-SIGN is a non-recycling receptor, and expression decreases after ligand internalization or maturation of DCs [38]. We also checked whether moDC maturation upon TLR stimulation was affected by ApoEV or ApoEV-HM loading, which would not be favorable in a therapeutic setting. The maturation capacity of LPS was not inhibited in ApoEV-loaded moDCs, as maturation marker expression was comparable between the different conditions (Figure 4B). This was in line with the results observed in a mixed-leukocyte reaction (MLR), where DC-induced CD4^+^ T cell and CD8^+^ T cell proliferation did not differ between the two conditions (Figure S5). Together, our results indicate that bortezomib-induced ApoEVs derived from Mel-JuSo cells do not have moDCs maturing properties, nor did they negatively impact moDC maturation upon TLR4 stimulation.

### 2.5. ApoEV-HM-Loaded moDCs Induce MART-1-Specific CD8^+^ T Cells

**Figure 5:**
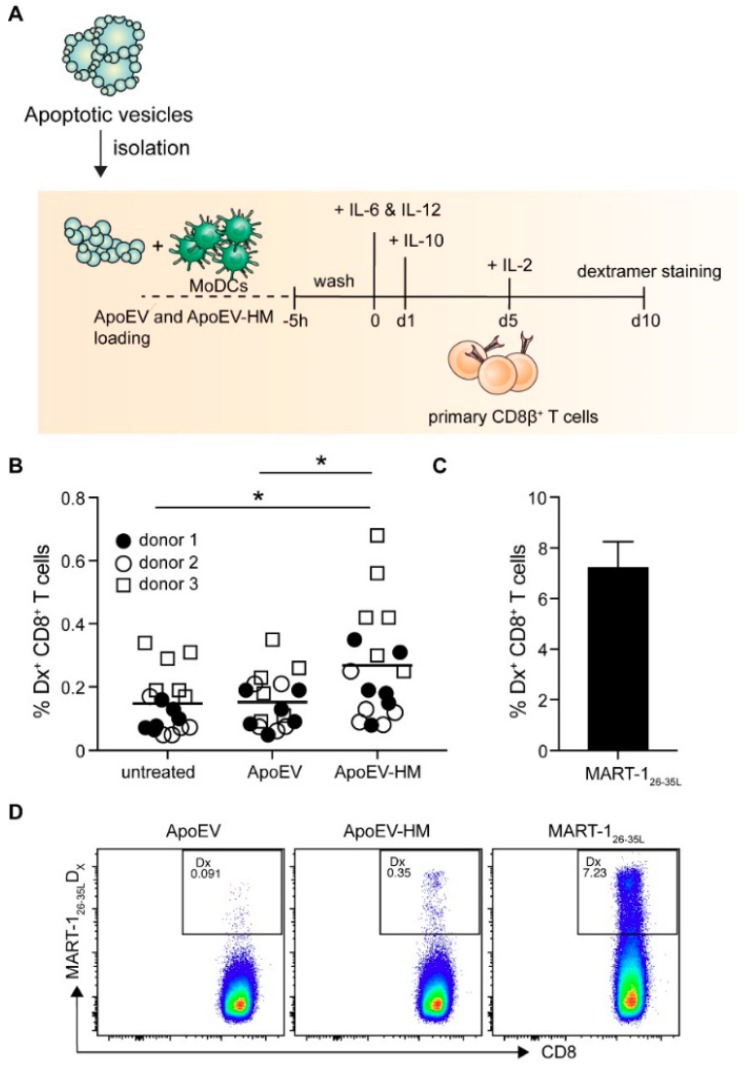
Glycan modification of ApoEVs (ApoEVs-HM) results in priming and expansion of MART-1-specific CD8^+^ T cells. (**A**) Protocol of priming and expansion of MART-1-specific CD8^+^ T cells. (**B**) MoDCs were loaded with the different vesicles in the presence of LPS. For the first and third donor six, and for the second donor five separate cultures were evaluated for each condition. Frequencies of MART-1_26–35L_-dextramer^+^-CD8^+^ T cells were analyzed 10 days after induction. Combined data of three donors is shown as percentage of MART-1_26–35L_-dextramer^+^-CD8^+^ T cells. Data were analyzed by one-way ANOVA with Tukey post-hoc test, * *p* < 0.05. (**C**) Frequencies of MART-1_26–35L_ Dx + CD8^+^ T cells present in the control cultures using the short MART-1_26–35L_ peptide. Data is shown as mean + SD of donor 1. (**D**) Representative flow cytometry plots of MART-1_26–35L -_Dx^+^ CD8^+^ T induced by ApoEV-, ApoEV-HM-, or short peptide (control)-loaded moDCs.

Finally, we assessed if the increased uptake of ApoEVs-HM also resulted in the cross-priming of tumor-associated antigen-specific HLA-A2-restricted CTLs. The ApoEVs were isolated from HLA-A2^−^ Mel-JuSo cells in order to prevent direct HLA-A2-restricted antigen presentation of the ApoEVs to T cells. To determine the potency of ApoEV-HM-loaded moDC to prime naïve T cells, we used the MART-1 (/Melan-A) as a melanoma-specific antigen. Since endogenous MART-1 was not detected in Mel-JuSo cells, we transduced the cells with MART-1. ApoEVs and ApoEVs-HM derived from the MART-1-expressing Mel-JuSo cell lines contained MART-1 protein, which we confirmed by Western blot (Figure S6). CD8β^+^ T cells were co-cultured with autologous irradiated CD8β^+^ depleted PBMCs (feeders) and allogeneic, HLA-A2-matched vesicle-loaded moDCs for 10 days (Figure 5A). As a read-out, we used dextramers (Dx), recognizing the high-affinity altered-peptide ligand for the immune-dominant MART-1 epitope MART-1_26–35_, i.e., MART-1_26–35L_ [40]. MART-1-specific CD8^+^ T cells could be detected at higher frequencies in cultures stimulated with moDCs loaded with ApoEVs-HM compared to cultures containing the wild-type ApoEV-loaded moDCs (Figure 5B,D). As a positive control, moDCs were pulsed with a short synthetic peptide of the immune-dominant MART-1 epitope MART-1_26–35L_ (Figure 5C,D), and unpulsed moDCs (no ApoEVs) were taken along as negative control. ApoEVs-HM induced higher percentages of MART-1-specific CD8^+^ T cells compared to the wild-type ApoEVs, thereby demonstrating that increased internalization of apoptotic vesicles by moDCs results in enhanced CD8^+^ T cell priming capacity.

In conclusion, we provide evidence that glycan modification of ApoEVs promotes vesicle internalization and CD8^+^ T cell priming.

## 3. Discussion

Targeting tumor-specific antigens to DCs has been shown to increase tumor vaccination efficacy in murine in vivo models [9,13]. However, most of these studies used single antigen formulations, whereas multi-antigenic vaccines hold the power to elicit more (poly)clonal T cell responses directed against multiple tumor-associated (neo-)antigens, thereby broadening anti-tumor immunity and avoiding immune escape. In this study, we investigated multi-antigenic glycan modified ApoEVs as a DC-targeting vaccine to enhance anti-tumor immunity. Our data show that the applied glycan modification of ApoEVs (high mannosylation) resulted in robust targeting to the CLR DC-SIGN. Moreover, it enhanced vesicle uptake and facilitated efficient tumor-specific CD8^+^ T cell priming, without altering the moDC phenotype or interfering with TLR-mediated DC maturation.

The induction of high-mannose glycans on ApoEVs facilitates efficient DC-SIGN-mediated antigen loading of DCs. The increase in uptake could be fully abrogated by blocking DC-SIGN, thereby excluding a role for the MR. A similar effect was previously observed regarding the uptake of high-mannose gp100 glycoproteins, which were also solely internalized via DC-SIGN [19]. The binding and internalization of ApoEVs did not result in the expression of maturation markers, which was in line with our previous findings showing that heat shock-induced leukemia-derived ApoEVs had no maturing effect on moDCs [26]. However, contradictory data have been published, where uptake of ApoEVs induced upregulation of DC maturation markers (e.g., CD40 and CD86) and secretion of pro-inflammatory cytokines (e.g., IL-6 and TNF-α) by murine bone marrow-derived dendritic cells (BMDCs), and the expression of CD80, CD83 and CD86, and secretion of IL-8 and TNF-α by human moDCs [41,42]. As ApoEVs are not well characterized and the isolation methods differ greatly between studies, these variations in DC-activating effects may be explained by the differences in vesicle origin, the timing of administration, the methods used for apoptosis induction, the isolation of the vesicles, or the size of the vesicles. The effect of vesicle size on immune activation was emphasized by the observation that ApoEVs (size 1–3 μM) derived from murine endothelial cells contained the pro-inflammatory cytokine IL-1α, while the smaller vesicles (<1 μM) did not [43]. When injected in the peritoneal cavity of mice, the larger ApoEVs (1–3 μM) induced a sterile inflammation with neutrophil infiltration [43]. Nevertheless, apoptotic cells can also have an immune inhibitory effect. Apoptotic cell uptake by TLR-stimulated DCs has been reported to result in the inhibition of NF-κB activation, reduced secretion of the pro-inflammatory cytokine IL-12, hampered CD86 expression and the secretion of the regulatory cytokine IL-10 [44,45,46]. In contrast, our isolated ApoEVs did not inhibit the upregulation of DC maturation markers (CD80, CD83, CD86, and HLA-DR) upon TLR-stimulation, and no differences were observed in the capacity to induce T cell proliferation between maturing ApoEV-loaded and non-ApoEV-loaded DCs in vitro. This is not surprising, as we have seen before that matured moDCs retain their capacity to express CCR7 and migrate towards CCL19 after loading with ApoEVs [26]. Fransen et al. showed an increase in T cell-mediated production of IL-2, IFN-gamma, and, in particular, IL-17 induced by ApoEV-loaded BMDCs. However, their mouse-derived (32Dcl3 cells) ApoEVs had BMDC maturing properties and during their in vitro experiments, no additional adjuvant was administered [41].

Multiple studies have already reported on the ability of ApoEV-loaded DCs to induce antigen-specific CD8^+^ T cell responses [26,27,47]. Our unmodified ApoEVs were not able to induce robust MART-1_26–35_-specific CD8^+^ cell responses. The amount of antigen expressed by the tumor cells, the immunogenicity of the model antigen, and the experimental setup are potential factors influencing T cell priming by ApoEVs. The work of Muhsin-Sharafaldine et al. demonstrated that ApoEVs from B16 tumors endogenously expressing ovalbumin did not result in anti-tumor protection, while after antigen enrichment, ApoEVs were superior in the induction of anti-tumor immunity compared to antigen enriched exosomes and micro-vesicles [47]. We did observe a significant increase in the MART-specific CD8^+^ T cells frequency in the primed cultures, where moDCs were loaded with glycan-modified ApoEVs, indicating that the enhanced DC-SIGN-mediated uptake augments T cell responses. These results verify the concept that DC-SIGN targeting, via Abs, glycan-modified liposomes, and modified ApoEVs, can boost CD8^+^ T cell responses [9,10,38].

Future research should focus on the minimum antigen content of ApoEVs necessary for the induction of strong anti-tumor T cell responses, preferably in an in vivo setting, using neo-antigens or non-self-antigens as model antigens. Additionally, inclusion of multiple neo-antigens in the vaccine is essential, given that the expression of neo-antigens can be selectively lost in tumors and may lead to tumor resistance [48]. Using patient-derived glycan-modified vesicle vaccines could therefore be an abundant and easily accessible source for tumor-specific antigens that circumvents the need for neo-antigen characterization and HLA matching. The content of these vesicles, and the effect of different levels of various (neo-)antigens present in these vesicle preparations, should be assessed to appreciate their impact on the induced anti-tumor immune response and the ultimate vaccination and anti-tumor efficacy achieved.

## 4. Materials and Methods

### 4.1. Reagents and Antibodies

The following reagents were used: Saponin (Sigma-Aldrich, Zwijndrecht, The Netherlands), monophosphoryl lipid A (MPLA) from *Salmonella enterica* (InvivoGen, Toulouse, France), lipopolysaccharide (LPS) from *Escherichia coli* (Sigma-Aldrich) Staphylococcal enterotoxin B from Staphylococcus aureus (SEB) (Sigma-Aldrich), Phytohemagglutinin-L (PHA-L) (Vector laboratories, Burlingame, CA, USA), paraformaldehyde (PFA) aqueous solution (Electron Microscopy Sciences, Hatfield, PA, USA), citric acid (Merck Millipore, Amsterdam, The Netherlands), sodium acetate (J.T Baker), TMB (Sigma-Aldrich), 2-mercaptoethanol (Sigma-Aldrich), Laemmli buffer (Bio-Rad, Veenendaal, The Netherlands), bovine serum albumin (BSA) (Roche, Nederland BV, Woerden, The Netherlands), trypsin (ThermoFisher Scientific, Breda, The Netherlands), bortezomib (Selleck, Munich, Germany), human serum (Sigma-Aldrich), human Interleukin-2 (IL-2) (Proleukin (Novartis), Arnhem, The Netherlands), IL-4 (ImmunoTools, Friesoythe, Germany), human IL-6 (R&D Systems, Minneapolis, MN, USA), human IL-10 (R&D Systems), human IL-12 (Peprotech, London, UK), human granulocyte-macrophage colony-stimulating factor (GM-CSF) (ImmunoTools), Kifunensine (Tocris Bioscience, Abingdon, UK) and Bradford protein assay kit (ThermoFisher). The following fluorescent labeled probes and Abs were used: PO-labeled Goat anti-human IgG Fc (Jackson, Ely, UK), anti-CD8β (clone 2ST8.5H7, Beckman Coulter, Brea, CA, USA), anti-MART-1 (clone M2-7C10, ThermoFisher), rabbit anti-GAPDH (14C10, Cell Signaling, Leiden, The Netherlands), anti-mouse-800 (LI-COR Biosciences, Lincoln, NE, USA), anti-rabbit-680 antibodies (LI-COR Biosciences), FITC-labeled anti-human IgG-Fc (Jackson), anti-CD80-FITC (clone 2D10, Biolegend, San Diego, CA, USA), anti-CD83-PE-Cy7 (clone HB15e, eBioscience, Santa Clara, CA, USA), anti-CD86-PE (clone 2331 (FUN-1), BD Biosciences, Temse, Belgium), anti-HLA-DR-BV510 (clone G46-6, BD Biosciences), anti-CD1a-FITC (clone HI149, BD Biosciences), anti-CD14-PE (clone M5E2, Biolegend), anti-EpCAM-BV421 (clone EBA-1, BD Biosciences), unconjugated anti-CD206 (clone 19.2, BD Biosciences), anti-CD8-BV421 (clone RPA-T8, BD Biosciences), anti-CD4-APC (clone RPA-T4, BD Biosciences), streptavidin-PO (Biosource), AF-488-streptavidin (Invitrogen), Violet tracer proliferation kit (ThermoFisher), FITC-Annexin V (BD Biosciences), the monoclonal IgG1 Ab AZN-D1 and AF488 labeled AZN-D1, against the carbohydrate-recognition domain of DC-SIGN (in house production) [35], PE-labeled MART-1_26–35L_ dextramer (MART-11_26–35L_ Dx, Immundex, Copenhagen, Denmark), the lipophilic tracer DiD, and the fixable viability dye (FVD) eFluor 780 (both from Invitrogen). The goat-anti-mouse (GaM) magnetic beads and human CD14 beads came from Miltenyi Biotec (Bergisch Gladbach, Germany).

### 4.2. Cell Lines, Lentiviral Transduction, Glycan Modification, and Apoptosis Induction

The human HLA-A2 negative (HLA-2^−^) melanoma cell lines Mel-JuSo and SK-MEL28 (a gift from prof. T.D. de Gruijl, Amsterdam UMC, Amsterdam) were cultured in IMDM medium (Invitrogen) supplemented with 10% FCS, 100 U/mL penicillin/streptomycin, and 2 mM l-glutamine (all from Lonza, Verviers, Belgium) (complete RPMI). For the generation of the MART-1-expressing Mel-JuSo cells, the MART-1 gene (a gift from prof. B.J. van den Eynde, Ludwig Institute, Brussels) was cloned using the NheI and XbaI sites of the PMEL human lentiviral vector (ABM, cat. no. LV801443, accession number NM_006928). An empty control vector was generated by removal of the PMEL human gene using EcoRV5 and subsequent re-ligation. Recombinant lentiviruses were produced by co-transfecting sub-confluent human embryonic kidney (HEK) 293T cells with the lentiviral expression plasmid and packaging plasmids (pMDLg/pRRE and pRSV/Rev) using calcium phosphate as transfection agent. HEK 293T cells were cultured in complete DMEM, in a 37 °C incubator with 5% CO_2_. Infectious lentivirus was collected 48 h after transfection and the supernatant was centrifuged to remove cell debris and stored at −80 °C. Mel-JuSo cells were transduced with the lentivirus preparations and after 48 h, cells expressing MART-1/GFP were selected by puromycin (0.2 × 10^−7^ g/mL) containing selection medium. Three days before induction of apoptosis (day 3), the Mel-JuSo culture medium was supplemented with 2 μg/mL kifunensine. At day 0, 4 × 10^6^ Mel-JuSo cells were seeded in T75 flask (10 mL), supplemented with 2 μg/mL kifunensine. The cells were allowed to adhere for at least 2 h and apoptosis was induced by adding 20 nM bortezomib to the culture medium for 72 h. Cell viability and apoptotic rate was assessed via staining with FITC-labeled Annexin V and fixable viability dye. In short, the culture medium and adherent cells, after trypsinization, were pooled and stained with FVD for 30 min. Hereafter, cells were washed twice and stained with FITC-labeled Annexin V in Annexin V staining buffer for 15 min at RT. Cells were washed and resuspended in Annexin V staining buffer and analyzed by flow cytometry on a ×20 Fortessa SORP flow cytometer (BD Biosciences).

### 4.3. ApoEV Isolation Protocol

The vesicles were collected 72 h after the induction of bortezomib-induced apoptosis (4.2.) via differential centrifugation steps: 10 min at 400× *g*, 20 min at 1200× *g*, and 30 min 10,000× *g* (ApoEVs) in order to remove cell debris (400× *g* fraction) and larger ApoEVs (1200× *g* fraction) [32,33]. All centrifugation steps were performed at 4 °C. The pellet containing ApoEVs (10,000× *g* fraction) was used for further experiments. Protein concentration was determined by Bradford protein assay kit according to manufacturer’s instructions. Protein concentrations in the ApoEV and ApoEV-HM lysed isolated fractions were generally comparable. Size distribution of the ApoEVs and ApoEVs-HM were analyzed by nanoparticle tracking analysis (NTA, Nanosight,).

### 4.4. Monocyte-Derived DCs

Immature moDCs were generated from monocytes obtained from human peripheral blood mononuclear cells (PBMCs), isolated from buffy coats of healthy donors (Sanquin, Amsterdam, The Netherlands) by a sequential lymphoprep (Axis-Shield, Dundee, UK) and percoll (Amersham, GE Health care, Eindhoven, The Netherlands) gradient. The monocytes were then cultured for 5–6 days in complete RPMI in the presence of recombinant human GM-CSF (800 U/mL) and IL-4 (500 U/mL).

### 4.5. Lectin Binding Assays

Cells were cultured in T75 flasks and supplemented with kifunensine (2 µg/mL) for 3 days. At day 3, the cells were stained with DC-SIGN-Fc and the plant lectin *Narcissus pseudonarcissus* agglutinin (NPA). DC-SIGN-Fc was produced from transfectants as previously described [49]. After trypsinization, cells were washed twice in TSM buffer (20 mM Tris, pH 7.4, 150 mM NaCL, 1 mM CaCL_2_, 2 mM MgCL_2_) supplemented with 1% BSA and stained with biotinylated *Narcissus pseudonarcissus* agglutinin (NPA) (specific for α1–6 polymannose) and purified DC-SIGN-Fc (10 µg/mL) for 30 min at RT. Lectin binding was detected with AF488-conjugated Streptavidin and FITC-labeled anti-human IgG-Fc, viability of the cells was determined with FVD and cells were analyzed by flow cytometry. For lectin binding analysis on ApoEVs, ELISA plates (Nunc maxisorp, ThermoFisher) were coated o/n at 4 °C with 0.05–50 µg/mL ApoEVs in coating buffer (50 mM Na_2_CO_3_, pH 9.7). Plates were washed in TSM buffer containing 0.05% Tween and blocked with 1% fatty acid free BSA in TSM for 30 min at 37 °C. Plates were incubated with 50 µL DC-SIGN-Fc (2 µg/mL) or biotinylated NPA (1 µg/mL) for 1 h at RT. Binding was detected using PO-labeled Goat anti-human IgG Fc or streptavidin-PO (30 min on RT). Binding was quantified by the addition of substrate buffer (0.1 M citric acid, 0.1 mM sodium acetate, pH 4, 100 µg/mL 3,3′-5,5′-tetramethylbenzidine (TMB) and 0.006% H_2_O_2_. Absorbance was measured at 450 nm using a spectrophotometer (Bio-Rad).

### 4.6. Binding and Uptake of ApoEVs

ApoEVs were labeled with 0.5–1 μM DiD for 15 min at 37 °C. After labeling, vesicles were washed twice in PBS and centrifuged at 10,000× *g* for 30 min. Blocking DC-SIGN and MR was done by pre-treating the DCs with AZN-D1 (20 μg/mL), anti-MR (CD206) (40 μg/mL), or isotype control (40 μg/mL) for 20–30 min at 37 °C. Immature or maturing (10 µg/mL MPLA) moDCs (10 × 10^4^ per time point) were loaded with 50 μg/mL DiD labeled ApoEVs or ApoEVs-HM for 45 min on ice to allow binding and thereafter transferred to 37 °C. At different time points (0, 30 and 60 min) cells were washed, and subsequently fixed with 1% PFA. The cells were analyzed by flow cytometry (×20 Fortessa SORP flow cytometer, BD Biosciences).

### 4.7. In Situ Dermal Dendritic Cell Targeting

Human abdominal skin explants from healthy donors (Bergman Clinics, Bilthoven, The Netherlands) were injected intradermal with 20 μL serum free IMDM (supplemented with penicillin streptomycin, glutamine and gentamycin), containing 50 μg/mL ApoEVs or ApoEVs-HM. At the site of injection, a blister would appear, of which a punch biopsy (8 mm; Microtec) surrounding the blister was taken. Around 8 biopsies per condition were obtained and cultured in a 48-well plate, with the epidermis facing upwards. After 2 days, the crawl-out cells were harvested and stained for analyses by flow cytometry. To distinguish the different migrated dermal DC subsets, we stained with the following antibodies: HLA-DR, CD1a, CD14, EpCAM, and FVD.

### 4.8. MoDC Maturation Assay

Immature moDCs were seeded in a 96-well U-bottom plate (Greiner) at a concentration of 50 × 10^3^ cells per well and loaded with the different vesicles (2 donors 200 µg/mL and 2 donors 100 µg/mL or LPS (10 ng/mL)). After 3 h, moDCs were washed in PBS and cultured o/n. MoDCs were stained for the expression of DC maturation markers CD80, CD86, and HLA-DR for 30 min. Marker expression was measured by flow cytometry (×20 Fortessa SORP flow cytometer, BD Biosciences).

### 4.9. Mixed Leukocyte Reaction (MLR) and Cytokine Secretion

CD14^+^ monocytes were isolated from PBMCs by positive selection using anti-CD14 conjugated magnetics beads according to the manufacturer’s instructions. Monocytes were then cultured for 5 days in the presence of 500 U/mL IL-4 and 800 U/mL GM-CSF for the generation of immature moDCs. Peripheral blood lymphocytes (PBLs) from healthy donors were labeled with 5 μM violet tracer, according to the manufacturer’s instructions and co-cultured with allogeneic ApoEVs and ApoEVs-HM (200 μg/mL) loaded and LPS-stimulated (10 ng/mL) (24 h) moDCs in complete IMDM medium for 7 days. Control moDCs were stimulated with 5 ng/mL SEB or 5 μg/mL PHA-L. At day 7, cells were harvested and labeled with anti-CD4 and anti-CD8. Proliferation was determined using flow cytometry (×20 Fortessa SORP flow cytometer, BD Biosciences) and analyzed with FlowJo V10 software (Ashland, OR, USA).

### 4.10. Induction of MART-1-Specific CD8^+^ Effector T Cells

CD14^+^ monocytes and CD8β^+^ T cells were isolated from PBMCs of HLA-A2^+^ healthy donors by positive labeling with magnetic bead-labeled anti-CD14 or anti-CD8β antibody and goat-anti-mouse (GaM) magnetic beads (Miltenyi Biotec) using a magnetic cell-sorting device (Miltenyi Biotec), according to the manufacturer’s instructions. Next, monocytes were cultured for 5 days in in the presence of IL-4 and GM-CSF for the generation of immature moDCs. For the induction of MART-1-specific CTLs, 1 × 10^6^ CD8β^+^ T cells and 1 × 10^6^ irradiated (50 Gy) CD8β^−^autologous PBMCs were co-cultured with 1 × 10^5^ allogeneic HLA-A2^+^ ApoEV (200 μg/mL, 5 h), ApoEV-HM (200 μg/mL, 5 h), or MART_26–36L_ peptide-pulsed [40] and LPS-stimulated (10 ng/mL) moDCs for 10 days in Yssel’s medium [50] containing 1% human serum. The culture medium was supplemented with IL-6 (10 ng/mL) and IL-12 (10 ng/mL). Six (first en third donor) or five (second donor) equal cultures were initiated per condition. On day 1, 10 ng/mL IL-10 and at day 5, 25 international units (IU) of IL-2 were added to the culture medium. At day 10 the cells were collected and the CD8^+^ T cells were stained with PE-labeled MART_26–35L_-specific dextramers (Dx) for 20 min at 37 °C, after which the cells were stained for 30 min at 4 °C for CD8 expression and viability. The frequency of MART-1 specific CTLs was assessed by flow cytometry (×20 Fortessa SORP flow cytometer, BD Biosciences) and analyzed with FlowJo V10 software.

### 4.11. Imaging Flow Cytometry

MoDCs were pulsed for 45 min on ice with 100 μg/mL ApoEVs or glycan modified ApoEVs (ApoEVs-HM). Thereafter cells were washed and incubated at 37 °C for 0, 15, or 30 min, before fixation with 4% PFA. Images were acquired on the ImageStream ×100 (Amnis Corp., Seattle, WA, USA) and analyzed using Ideas v6.0 software (Amnis-Merck Millipore) as previously described [38]. In short, analysis was done on single cells after compensation on at least 300 cells/ time point for donor 1, 10,000 cells/time point for donor 2 and 2900 cells/time point for donor 3. The spot count was calculated on DiD^+^ cells by creating a mask based on the surface of cells in the bright field image. To calculate the internalization score, another mask was created and pixels were eroded from the mask to exclude the membrane. The resulting mask was applied to the fluorescence channel containing the probe of interest. The internalization score was calculated using features provided in the Ideas v6.0 software. DCs that have internalized the bound vesicles have positive scores, while DCs that have most vesicles located at the membrane have negative scores. If the internalization score is 0, equal amounts of vesicles are located at the cell surface as well as intra-cellular.

### 4.12. Western Blot

To prepare crude cell lysates, ApoEVs and ApoEVs-HM were washed and lysed with RIPA buffer (containing phosphatase and protease inhibitors) on ice. To prepare Western blot samples the lysate was boiled for 5 min at 95 °C in pre-heated 4× Laemmli buffer containing 5% 2-mercaptoethanol. Samples containing equal amounts of protein were loaded on a 15% polyacrylamide gel according to standard procedures. Nitrocellulose membranes (0.45 μm, Bio-Rad) were incubated o/n with mouse anti-MART-1 and rabbit anti-GAPDH. Anti-mouse-800 and anti-rabbit-680 antibodies were used as secondary antibodies. Membranes were analyzed using Odyssey Classic Imager (LI-COR) and Image Studio Lite (LI-COR).

### 4.13. Statistical Analysis

Data were analyzed with GraphPad Prism 7.0 software (La Jolla, CA, USA) using one-way ANOVA or two-way ANOVA with a post-hoc analysis as indicated in the figure legend. * *p* < 0.05, ** *p* < 0.01, *** *p* < 0.001.

## 5. Conclusions

In conclusion, mannosylated glycan-enriched melanoma ApoEVs target DC-SIGN on moDCs, thereby enhancing vesicle uptake, cross-presentation and the priming and activation of tumor-specific CD8^+^ T cells. DC-SIGN-binding patient-derived ApoEVs could therefore be a promising multi-antigenic source for DC-targeted anti-tumor immunotherapy.

## Supplementary Materials

The following are available online at https://www.mdpi.com/2072-6694/11/9/1266/s1, Figure S1: MoDCs favor ApoEVs–HM isolated at 10,000× *g*. MoDCs were incubated with DiD-labeled ApoEVs or ApoEVs-HM (50 μg/mL) isolated at 1200× *g* and 10,000× *g* for 45 min at 4 °C, before cells were transferred to 37 °C for 60 min. Uptake was measured by flow cytometry. Data shown as mean ± SD of *n* = 4. Data analyzed with one-way repeated measures ANOVA with Tukey post-hoc test, * *p* < 0.05. Figure S2: Profile of size distribution of ApoEVs and ApoEVs-HM, measured by nanoparticle tracking analysis (NTA). Figure S3: SK-Mel28-derived ApoEVs-HM express DC-SIGN binding ligands. Different concentrations of SK-Mel28-derived ApoEVs were coated on an ELISA plate and DC-SIGN binding was detected by DC-SIGN-Fc staining. Figure S4: Simultaneous administration of a TLR4-stimulus did not affect ApoEV-HM uptake. Internalization of ApoEVs-HM after 0, 30, and 60 min by TLR4-stimulated moDCs (10 μg/mL MPLA). Data of *n* = 2. Figure S5: Allogeneic mixed leukocyte reaction (MLR), ApoEV- and ApoEV-HM-loaded (200 μg/mL) and LPS-stimulated moDCs and violet tracer stained peripheral blood lymphocytes (PBLs) were co-cultured for 7 days. T cell proliferation was analyzed by flow cytometry using the violet tracer dilution as a read-out. Data represents the mean + SD and are representative graphs of *n* = 2. Figure S6: Western blot for the detection of MART-1 protein (18 kDa) in the ApoEVs and ApoEVs-HM. Equal amounts of protein were loaded of lysed ApoEVs and ApoEVs-HM.
