# Supplementary material for: Glycan-Modified Apoptotic Melanoma-Derived Extracellular Vesicles as Antigen Source for Anti-Tumor Vaccination
Source: Cancers (Basel). 2019 Aug 28;11(9):1266. doi: 10.3390/cancers11091266 (PMC6769957; doi:10.3390/cancers11091266)
Supplement: Supplementary file 1 [file cancers-11-01266-s001.pdf]

Supplementary Materials: Glycan-Modified Melanoma-Derived Apoptotic Extracellular Vesicles as Antigen Source for Anti-Tumor Vaccination

Sophie K. Horrevorts, Dorian A. Stolk, Rieneke van de Ven, Myrthe Hulst, Bert van het Hof, Sanne Duinkerken, Marieke H. Heineke, Wenbin Ma, Sophie A. Dusoswa, Rienk Nieuwland, Juan J. Garcia Vallejo, Arjan A. van de Loosdrecht, Tanja D. de Gruijl, Sandra J. van Vliet and Yvette van Kooyk

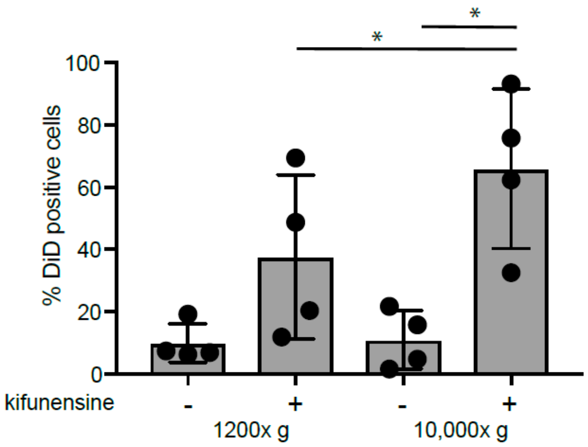

Figure S1. MoDCs favor ApoEVs–HM isolated at 10,000× *g*. MoDCs were incubated with DiD labeled ApoEVs or ApoEVs-HM (50 µg/mL) isolated at 1200× *g* and 10,000× *g* for 45 min at 4 °C, before cells were transferred to 37 °C for 60 min. Uptake was measured by flow cytometry. Data shown as mean ± SD of $n = 4$. Data analyzed with one-way repeated measures ANOVA with Tukey post-hoc test, * $p < 0.05$.

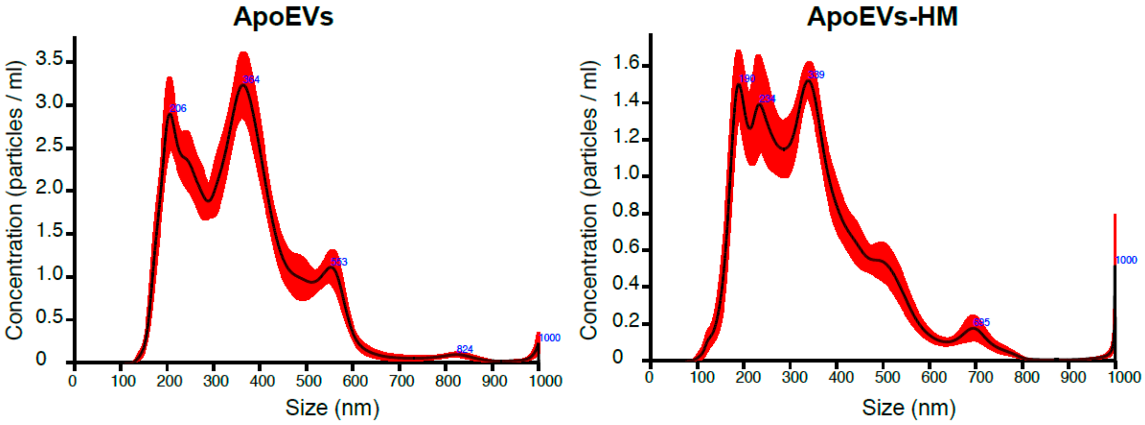

Figure S2. Profile of size distribution of ApoEVs and ApoEVs-HM, measured by nanoparticle tracking analysis (NTA).

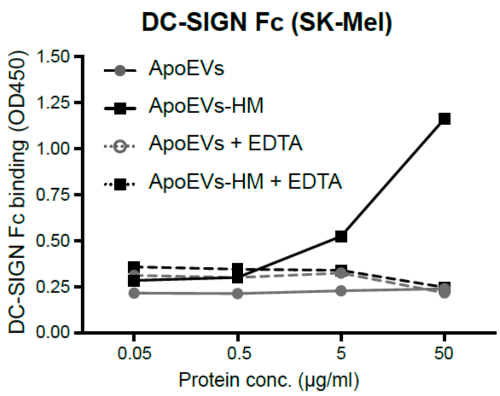

Figure S3. SK-Mel28 derived ApoEVs-HM express DC-SIGN binding ligands. Different concentrations of SK-Mel28-derived ApoEVs were coated on an ELISA plate and DC-SIGN binding was detected by DC-SIGN-Fc staining.

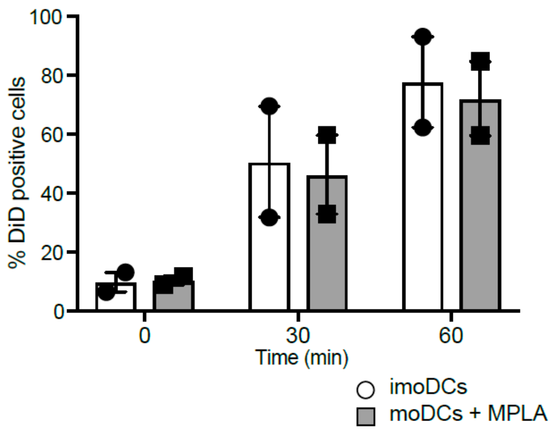

Figure S4. Simultaneous administration of a TLR4-stimulus did not affect ApoEV-HM uptake. Internalization of ApoEVs after 0, 30 and 60 min by TLR4-stimulated moDCs (10 µg/mL MPLA). Data of n=2.

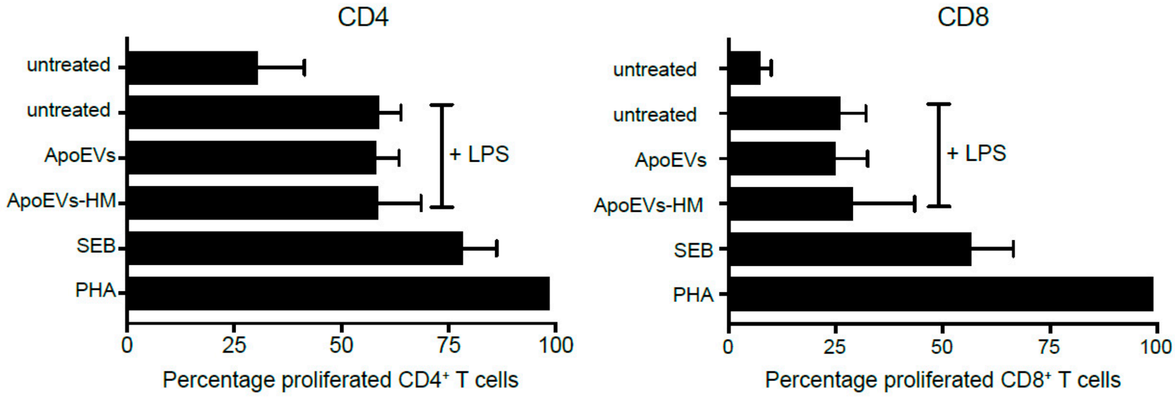

Figure S5. Allogeneic mixed leukocyte reaction (MLR), ApoEV- and ApoEV-HM-loaded (200 µg/mL) and LPS-stimulated moDCs and violet tracer stained peripheral blood lymphocytes (PBLs) were co-cultured for 7 days. T cell proliferation was analyzed by flow cytometry using the violet tracer dilution as a read-out. Data represents the mean + SD and are representative graphs of *n* = 2.

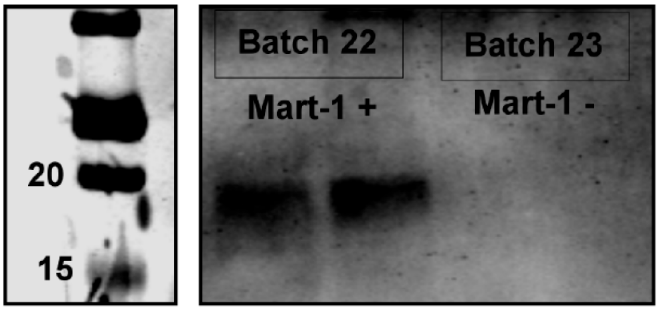

Figure S6. Western blot for the detection of MART-1 protein (18 kDa) in the ApoEVs and ApoEVs-HM. Equal amounts of protein were loaded of lysed ApoEVs and ApoEVs-HM.

© 2019 by the authors. Licensee MDPI, Basel, Switzerland. This article is an open access article distributed under the terms and conditions of the Creative Commons Attribution (CC BY) license (http://creativecommons.org/licenses/by/4.0/).
